# Psychometric Properties and Predictive Value of a Screening Questionnaire for Obstructive Sleep Apnea in Young Children With Down Syndrome

**DOI:** 10.3389/fpsyt.2020.00285

**Published:** 2020-04-28

**Authors:** Sarah Grantham-Hill, Hazel J. Evans, Catherine Tuffrey, Emma Sanders, Heather E. Elphick, Paul Gringras, Ruth N. Kingshott, Jane Martin, Janine Reynolds, Anna Joyce, Catherine M. Hill, Karen Spruyt

**Affiliations:** ^1^Faculty of Medicine, School of Clinical and Experimental Sciences, University of Southampton, Southampton, United Kingdom; ^2^Department of Sleep Medicine, Southampton Children’s Hospital, Southampton, United Kingdom; ^3^Department of Community Child Health, Solent NHS Trust, Portsmouth, United Kingdom; ^4^Department of Respiratory and Sleep Medicine, Sheffield Children’s NHS Foundation Trust, Sheffield, United Kingdom; ^5^Department of Sleep Medicine, Evelina London Children’s Hospital, Guys St Thomas’s NHS Trust, London, United Kingdom; ^6^Southampton Centre for Biomedical Research, University Hospital Southampton NHS Trust, Southampton, United Kingdom; ^7^School of Psychotherapy & Psychology, Faculty of Humanities, Arts and Social Sciences, Regent's University London, London, United Kingdom; ^8^Laboratoire de Physiologie intégrée du système d’éveil CRNL‐ INSERM U1028‐CNRS UMR 5292, Université Claude Bernard Lyon 1, Hospices Civils de Lyon, Lyon, France

**Keywords:** Down syndrome, trisomy 21, screening, obstructive sleep apnea/apnea, psychometric properties

## Abstract

**Study Objectives:**

Obstructive sleep apnea (OSA) is common in children with Down syndrome (DS) and is associated with adverse health and cognitive outcomes. Daytime clinical assessment is poorly predictive of OSA, so regular screening with sleep studies is recommended. However, sleep studies are costly and not available to all children worldwide. We aimed to evaluate the psychometric properties and predictive value of a newly developed screening questionnaire for OSA in this population.

**Methods:**

202 children aged 6 months to 6^th^ birthday with DS were recruited, of whom 188 completed cardio-respiratory sleep studies to generate an obstructive apnea hypopnea index (OAHI). Parents completed the 14-item Down syndrome OSA screening questionnaire. Responses were screened, a factor analysis undertaken, internal consistency calculated and receiver operator characteristic (ROC) curves drawn to generate an area under the curve (AUC) to assess criterion related validity.

**Results:**

Of 188 children who completed cardiorespiratory sleep studies; parents completed the screening questionnaire for 186. Of this study population 15.4% had moderate to severe OSA defined by an OAHI of ≥5/h. Sixty-three (33.9%) participants were excluded due to “unsure” responses or where questions were not answered. Using the remaining 123 questionnaires a four-factor solution was found, with the 1^st^ factor representing breathing related symptoms, explaining a high proportion of the variance. Internal consistency was acceptable with a Cronbach alpha of 0.87. ROC curves for the total score generated an AUC statistic of 0.497 and for the breathing subscale an AUC of 0.603 for moderate to severe OSA.

**Conclusion:**

A well designed questionnaire with good psychometric properties had limited predictive value to screen for moderate to severe OSA in young children with DS. The use of a screening questionnaire is not recommended. Screening for OSA in this population requires objective sleep study measures.

## Introduction

Down syndrome (DS) is the commonest chromosomal abnormality affecting approximately 1:1,200 live births worldwide (1). Children with DS are at increased risk of obstructive sleep apnea (OSA) a condition characterized by repetitive partial (hypopnea) or complete (apnea) airway collapse in sleep, despite continued respiratory effort. OSA is estimated to affect 75%, of this population compared to 1.2% of typically developing (TD) children (2, 3). Risk factors are multifactorial including syndrome-specific characteristics such as hypotonia, macroglossia, craniofacial structure, and obesity, exacerbated by adenotonsillar hypertrophy in early childhood (4).

OSA causes nocturnal hypoxia and fragmented sleep with adverse health consequence that have been extensively studied in TD children including: hypertension (systemic and pulmonary) (5), cognitive deficits (impaired attention and executive function) leading to impaired learning and school performance (6), as well as reduced quality of life (7), and increased health care utilization (8). Similar findings are emerging in children with DS who arguably may be more at risk due to their limited cognitive reserve and underlying cardiovascular disease (9). Indeed, Breslin et al.. studied 38 school-aged children with DS and reported that co-occurring OSA was associated with a nine point reduction in verbal IQ and reduced cognitive flexibility (10). We have also recently reported that OSA predicts deficits in parent-reported executive function behaviors in very young children with Down syndrome (11). It has further been hypothesized that OSA in DS may be a risk factor for the development of Alzheimer’s disease (12). Prompt identification and treatment of OSA in DS is therefore an important goal.

Multiple studies have reported poor correlation between parental report of OSA symptoms and polysomnography (PSG) results (the gold standard for the diagnosis of OSA) (13, 14). This may be due to a lack of awareness of nocturnal symptoms or the presence of silent apnea which is difficult for parents to detect. Children with DS referred for PSG have more severe disease than TD children, suggesting that milder symptoms are overlooked or attributed to unmodifiable symptoms of DS.

Given the increased burden of disease and challenges in diagnosis in this population, the American Academy of Pediatrics recommends routine screening with PSG by the age of 4 years (15). There is evidence of limited compliance with these guidelines with one study reporting that only 47.7% of children had undergone a PSG (16). In the UK, screening is recommended annually from infancy to 3–5 years of age in DS, using a minimum of pulse oximetry (17). If there is any abnormality detected on pulse oximetry, or clinical suspicion of a false negative oximetry result, then further assessment with, as a minimum, cardiorespiratory polygraphy studies is recommended (17). We have published recommended oximetry screening thresholds that can be used to determine the need for further diagnostic evaluation in this population (18). This screening method has a high reported sensitivity (92%) with a specificity (63%) with one night of domiciliary Masimo pulse oximetry. While oximetry is a screening tool that is, on the whole, well tolerated, it has resource implications (19). Other groups have researched alternative screening methods, including urinary biomarkers, 3D photogrammetry, and combined measures including cephalometry and multiple clinical variables (20–22). All of these approaches have limitations of time and cost and therefore a screening questionnaire is an appealing alternate approach.

Screening questionnaires are used in clinical practice to identify sleep problems in TD children. There has been increasing work looking at the utility of these questionnaires in the DS population. Ebsensen et al.. studied the convergent validity of three questionnaires, the Behavioral Evaluation of Disorders of Sleep (BEDS), Children’s Sleep Habits Questionnaire (CSHQ), and Sleep Disturbances Scale for Children (SDSC) in a group of 30 children with DS aged 6–17 years. All three questionnaires have sub-scales relating to sleep disordered breathing and were previously validated (23–25). There were strong correlations between these sub-scales but, in the absence of an objective measure of OSA in this study, no conclusions could be drawn about the sub-scales’ ability to predict OSA (26). OSA screening questionnaires have been designed for TD children. The Pediatric Sleep Questionnaire (PSQ) had initial reported sensitivities and specificity of 0.85 and 0.87 respectively to predict moderate to severe OSA in TD children at 2–18 years (27, 28), however concerns have been raised about its specificity within TD populations. Sproson et al.. reported specificity of only 0.17 for an OAHI ≥5/h in a young UK population (29). It may have further limitations in the DS population as it includes questions that relate to child behavior and growth that may be due to underlying features of their DS, as opposed to co-occurring OSA. Cielo et al.. encountered this difficulty when testing the PSQ in children with cranio-facial abnormalities where sensitivity and specificity were only 0.57 and 0.48 respectively to predict moderate to severe OSA (30). Furthermore, Pabery et al.. reported an even lower sensitivity of 0.37 for the PSQ to predict moderate to severe OSA in 35 children with Down syndrome aged 2–16 years (31).

We have previously reported the methodology used to design a 14-item OSA screening questionnaire intended for children with DS aged up to 6 years (32, 33). Specifically, we used a content validity process to design a questionnaire specific to children with DS incorporating expertise from health care professional and parents into the design process. Details of this process are outlined elsewhere (28, 29). The present study aimed to evaluate the psychometric properties and predictive value of this questionnaire when tested in a population of young children with DS.

## Materials and Methods

### Participants

Children with a confirmed diagnosis of DS between the ages of 6 months to 6^th^ birthday were recruited to one of three research centers in the UK at Southampton, Sheffield, and The Evelina London Children’s hospitals. Children were excluded if they had undergone a cardiorespiratory sleep study in the preceding 3 months, were receiving home oxygen therapy or non-invasive ventilation. Children were recruited through multiple approaches as previously described (19).

### Measures

#### Demographics and Medical History

Parent/caregivers provided information on their child’s age, gender, relevant past medical history (use of prophylactic asthma treatment, upper airway surgery, epilepsy, congenital cardiac condition, home oxygen use and whether born prematurely under 37 weeks gestation) and socio-demographic characteristics including parental education levels and smoking status. Children were weighed and measured and a body mass index calculated.

#### Questionnaire

The DS OSA questionnaire, developed by Sanders et al. (32), comprises 14 items rated on a five point Likert scale: never (never in the past 6 months), Rarely (less than one night a week), Occasionally (1–3 nights a week), almost always (4–6 nights a week), always (every night). An additional “unsure” response was allowed for each item. The questionnaire was designed to be completed by the child’s primary caregiver. Details of the questions can be found in **Tables 1** and **4**.

**Table 1:** Number of missing and unsure data items as per question response (N=186).

Question number	Missing data items [frequency (%)]	Unsure data items [frequency (%)]
1. How often does your child snore when they do not have a cold?	0 (0.0%)	4 (2.2%)
2. How often can you hear your child snoring from outside the bedroom?	0 (0.0%)	6 (3.2%)
3. How often does your child struggle to breath while asleep?	1 (0.5%)	22 (11.8%)
4. How often does your child’s breathing go quiet and then he/she gasp?	0 (0.0%)	21 (11.3%)
5. When your child is asleep, how often do you touch/nudge your child to make them breathe again?	0 (0.0%)	11 (5.9%)
6. How often does your child sleep in unusual positions?	1 (0.5%)	1 (0.5%)
7. How often does your child have restless sleep?	2 (1.0%)	5 (2.7%)
8. How often does your child sweat while asleep?	1 (0.5%)	10 (5.4%)
9. How often does your child wake up during the night? (more than children of a similar age)?	1 (0.5%)	5 (2.7%)
10. How often does you child have difficulty waking up in the morning, even after getting plenty of sleep?	1 (0.5%)	1 (0.5%)
11. How often is your child grumpy first thing in the morning?	1 (0.5%)	2 (1.1%)
12. How often does your child tend to breathe during their mouth during the day?	0 (0.0%)	41 (22.0%)
13. How often is your child unusually sleepy during the day?	0 (0.0%)	8 (4.3%)
14. How often does your child appear more hyperactive or fidgety than children of a similar age?	0 (0.0%)	13 (7.0%)

#### Domiciliary Cardiorespiratory Polygraphy

OSA was assessed using the SOMNOtouch device (SOMNOmedics, Germany) comprising chest and abdominal respiratory inductance plethysmography (RIP) bands, internal pulse oximetry, nasal pressure flow with snore sensor, body position sensor, and actigraphy. We have previously reported our positive experience of domiciliary studies in this population (34). A sleep log recorded sleep onset, night waking’s, and morning wake up times.

Studies were scored by an experienced technologist (RNK), using Domino Light software (SOMNOmedics, Germany). Details of scoring criteria and quality assessment of studies have been published (19). Sleep and wake were estimated using parental sleep log and integrated actigraphy. As per AASM scoring criteria, where two or more signals were of poor quality, data were excluded. Respiratory events were scored according to standard pediatric scoring criteria for adapted sensors (35). Where nasal ﬂow signal was lost an “undeﬁned apnea” was scored, where RIP sum indicated paradoxical breathing in the presence of a minimum three percent oxyhemoglobin desaturation for at least two breaths. The obstructive apnea/hypopnea index (OAHI) was calculated by summing obstructive apnea, hypopnea, mixed and undefined apnea indices during the total sleep time. OSA was diagnosed if OAHI was ≥ 5/h representing both a meaningful threshold for clinical intervention and reflecting the sensitivity of domiciliary cardiorespiratory polygraphy in children (6).

### Procedure

The study was approved by the UK National Research Ethics Committee (reference 13/SC/0106). Parents provided informed consent for their child to participate. Procedures for the full study are published (18).

### Statistical Analysis

Analyses were performed using SPSS (IBM SPSS, version 22.00, Chicago, IL, USA). Questionnaire data were checked for entry error and 10% were double entered at random to check for data integrity.

Responses were initially screened for missing and unsure responses. Items at this point were considered for ongoing inclusion in further analysis. As principle component analysis and reliability analysis require continuous data entry, 63 participants who had given an “unsure” response or had failed to respond to any item were excluded.

Demographics, past medical history and OAHI were compared between the excluded and included participants using either the chi-squared test or paired T tests to detect significant differences between the groups.

Question responses were split into positive or negative responses based on clinical significance. For example, in question 1 “how often does your child snore when they do not have a cold” a negative response was counted as “never, rarely, and occasionally” and a positive response as “almost always and always.” Demographics and past medical history of participants were compared for these dichotomized responses using a chi-squared test or paired T-test to identify any bias in response.

A principal component analysis was conducted to identify structure within the questionnaire and to determine the relationship of its underlying dimensions. A Kaiser-Meyer-Olkin (KMO) adequacy of sample measure was performed (36). To be an adequate sample a value of 0.5 is required. Factors were initially extracted using an Eigen-value greater than one. To aid extraction, scree plots and an approach with fixed number of factors were used. An orthogonal varimax rotation aids interpreting the factors to produce defined subscales. Factors were interpreted and assigned meaning by the authors SG-H, KS, and CH. Stability of the factors were checked by performing a split half method and ensuring similar results were achieved to those for the group as a whole. Cronbach alpha, a measure of internal consistency, was checked for the scale as a whole. Internal consistency of the subscales was then measured using a split half method.

Receiver operator characteristic (ROC) curves were generated for both the total score of the questionnaire and the underlying subscales as predicators of OSA status. Questionnaire responses were scored from 1 to 5, where 1=never and 5=always. Area under the curve (AUC) statistics were generated: An AUC of 0.5 indicates no predictive power and an AUC of 1 indicates perfect predictive power.

The study aimed to choose a point on the curve which maximized sensitivity over specificity to identify the maximal number of true positives based on the concept that the questionnaire would act as an initial screening tool rather than a diagnostic tool. This value would be out of total maximum possible points scored from the questionnaire.

## Results

**Figure 1:**
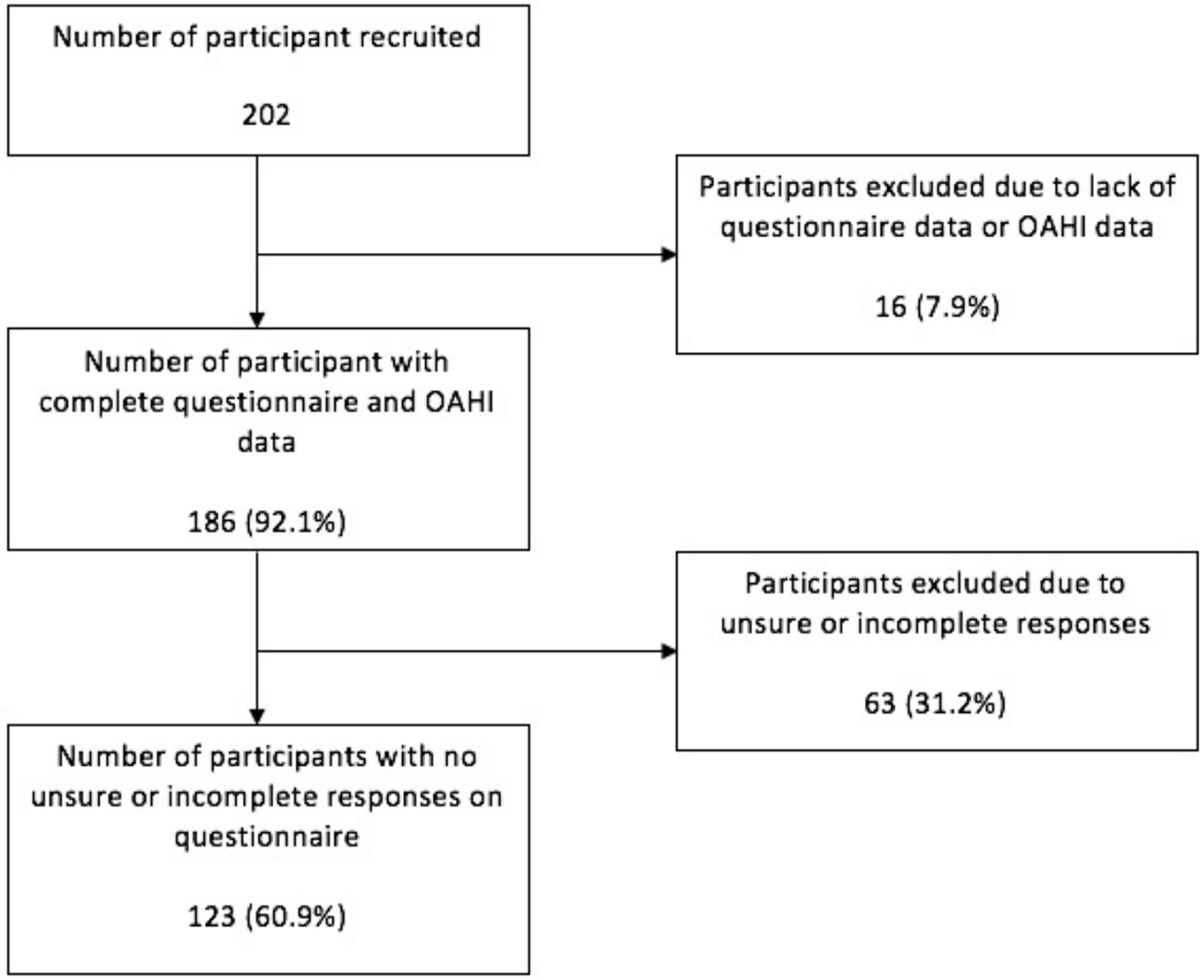
Participant flow throughout the study.

Two hundred two participants enrolled in the study, of whom 186 had both a completed DS OSA questionnaire and a calculated OAHI. Participant flow through the study is shown in **Figure 1**.

### Missing and Unsure Data Items

The number of unsure and missing data items among this sample of 186 are shown in **Table 1**. Twenty-two percent of all parents completing the questionnaire answered “unsure” to question 12 “how often does your child tend to breathe through their mouth during the day.” It was therefore felt to be a poor question and excluded from further analysis.

Sixty-three (33.9%) parents answered randomly “unsure,” or data were missing, for one or more other question and these questionnaires were excluded from data analysis leaving a sample of 123 (66.1%) fully completed questionnaires for the final analysis.

### Demographic and Clinical Characteristics

Demographics of the final sample are shown in **Table 2**. There were no significant differences in child’s age, gender, body mass index (BMI) category, relevant past medial history, parental socio-demographic features, or study center between the 123 (66.1%) participants included in the final analysis and 63 (33.9%) that were excluded. Significant difference in responses to questions are shown in **Table 3A** for when comparing “unsure response” to an alternative response and **Table 3B** when comparing dichotomized positive and negative responses to individual questions. Where significant differences in clinical or demographic characteristics were identified these were discussed by lead authors. It was agreed that these did not represent any systemic bias. No question items were rejected on the basis of these findings.

**Table 2:** Demographic and respiratory event differences for the included and excluded groups.

Item		Whole group (n=186)	Included (n=123)	Excluded (N=63)	P value
Gender	Male:female	99:87	65:58	34:29	0.504
Age in months (mean)		36.16 (SD 20.6) (range 6–71)	34.87 (SD 20.3) (range: 6–71)	38.68 (SD 21.1) (range 6–71)	0.659
**BM1 > 95^th^ centile [restricted to those aged ≥ 2 years, (N=129]**		24 (18.6%)	13 (16.8%)	11 (21.2%)	0.069
*Previous upper airway surgery*		33 (17.7%)	22 (17.8%)	11 (17.5%)	0.560
Parent 1 educational level	One GCSE at C level	29 (15.6%)	20 (16.3%)	9 (14.3%)	0.393
	A-level	23 (12.4%)	16 (13%)	7 (11.1%)	
	HND	35 (18.8%)	24 (18.5%)	11 (17.5%)	
	Degree	74 (39.8%)	50 (40.7%)	24 (38.1%)	
Parent 2 educational level	One GCSE at C level	22 (11.8%)	14 (11.4%)	8 (12.7%)	0.693
	A-level	23 (12.4%)	13 (10.6%)	10 (15.9%)	
	HND	29 (15.6%)	23 (18.7%)	6 (9.5%)	
	Degree	72 (38.7%)	48 (39%)	24 (38.1%)	
Respiratory event category	OAI > 1/h	44 (22.4%)	27 (22.0%)	17 (27%)	0.469
	OAHI≥2/h	81 (43.5%)	57 (46.3%)	24 (38.1%)	0.283
	OAHI≥5/h	26 (14%)	19 (15.4%)	7 (11.1%)	0.284
	OAHI≥10/h	14 (7.5%)	10 (8.1%)	4 (6.3%)	0.455

Further clarification of educational level: 1. GCSE at level C: has passed examinations conducted at the age of 16 years, 2. A-levels: has obtained examination results at the age of 18 years, 3. HND: post-18 year higher education achievement taken as alternative to a degree for more vocational subjects, 4. Degree: successful completion of a university course.

**Table 3A:** Responses to question items where proportion answering unknown varied significantly according to demographic and clinical characteristics of the sample (n=186).

Question item	Demographic or clinical characteristic		Number (%) or mean (SD) by item response		P value
			Response “unknown”	All other responses	
3. *How often can you hear your child struggle to breathe while asleep?*	History of upper airway surgery	Yes	0 (0%)	29 (17.8%)	0.018
		No	22 (100%)	134 (82.2%)
	Age (months)		42 (4.5%)	35 (1.6%)	0.032
4. *How often does your child’s breathing go quiet and then he/she gasps?*	BMI		17.87 (1.5)	16.82 (1.3)	0.03
13. *How often is your child unusually sleepy during the day?*	Smokers in the home	Yes	4 (50%)	17 (9.6%)	0.06
	No		4 (50%)	161 (90.4%)
14. *How often does your child appear more hyperactive of fidgety than children of a similar age?*	Smokers in the home	Yes	4 (30.8%)	17 (9.8%)	0.036
		No	9 (69.2%)	156 (90.2%)

Percentages are calculated out of respondents to the questions.

**Table 3B:** Demographic and clinical characteristics of the sample that differed significantly in respondents answering positively *versus* negatively to specific questions.

Question item	Demographic or clinical characteristic	Number (%) or mean (SD) by item response			P value
			Less positive response (Category 1)	More positive response (Category 2)	
1. *How often does your child snore when they do not have a cold?*	Presence of wheeze	Yes	51 (42.5%)	37 (59.6%)	0.014
		No	69 (57.5%)	24 (38.8%)	
		Not known	0 (0%)	1 (1.6%)	
	Use of prophylactic asthma treatment*	Yes	13 (10.9%)	12 (19.5%)	0.045
		No	40 (33.3%)	27 (43.4%)	
		Not applicable	67 (55.8%)	23 (37.1%)	
	Parental education level** Parent 1 Parent 2	Unknown	5 (4.4%) 13 (10.9%)	0 (0%) 7 (11.3%)	0.001 0.002
		No examinations	3 (2.7%) 2 (1.7%)	5 (8.1%) 6 (9.7%)	
		GCSE less than a D	5 (4.4%) 4 (3.4%)	5 (8.1%) 6 (9.7%)	
		GCSE more than a C	12 (10%) 11 (9.2%)	16 (25.2%) 11 (17.7%)	
		A levels	13 (10.9%) 13 (10.9%)	10 (16.2%) 10 (16.1%)	
		HND	21 (17.6%) 19 (16%)	14 (22.8%) 10 (16.1%)	
		Degree	60 (50%) 57 (47.9%)	12 (19.6%) 12 (19.3%)	
2. *How often can you hear your child snoring from outside the bedroom?*	Presence of wheeze	Yes	62 (43.4%)	24 (64.9%)	0.02
		No	81 (56.6%)	12 (32.4%)	
		Unsure	0 (0%)	1 (2.7%)	
	Parental education levels Parent 1 Parent 2	Unknown	5 (3.5%) 15 (10.6%)	0 (0%) 4 (10.8%)	0.001 0.002
		No examinations	6 (4.2%) 6 (4.2%)	2 (5.4%) 3 (8.1%)	
		GCSE less than a D	7 (4.9%) 8 (5.6%)	2 (5.4%) 2 (5.4%)	
		GCSE more than a C	16 (11.3%) 13 (9.2%)	12 (32.4%) 8 (21.6%)	
		A levels	17 (11.9%) 15 (10.6%)	6 (16.2%) 8 (21.6%)	
		HND	22 (15.5%) 21 (14.8%)	12 (32.4%) 8 (21.6%)	
		Degree	69 (48.7%) 64 (45.%)	3 (8.2%) 4 (10.8%)	
3. *How often can you hear your child struggle to breath while asleep?*	Presence of wheeze	Yes	40 (39.6%)	42 (67.7%)	0.001
		No	60 (59.5%)	20 (32.3%)	
		Unsure	1 (0.9%)	0 (0%)	
	Use of prophylactic asthma treatment	Yes	10 (9.9%)	15 (24.2%)	0.001
		No	32 (31.7%)	28 (45.2%)	
		Unapplicable	59 (58.4%)	19 (30.6%)	
	Parent 1 education	Unknown	4 (4%)	0 (0%)	0.001
		No examinations	1 (1%)	5 (8.1%)	
		GCSE less than a D	2 (2%)	6 (9.6%)	
		GCSE more than a C	13 (13%)	13 (21%)	
		A levels	11 (11%)	10 (16.1%)	
		HND	19 (19%)	14 (22.6%)	
		Degree	50 (50%)	14 (22.6%)	
4. *How often does your child’s breathing go quiet and then he/she gasps?*	Parental education level Parent 1 Parent 2	Unknown	4 (5.3%) 8 (10.4%)	1 (1.1%) 10 (11.6%)	0.001 0.003
		No examinations	1 (1.3%) 1 (1.3%)	6 (6.9%) 7 (8%)	
		GCSE less than a D	4 (5.3%) 3 (3.9%)	4 (4.6%) 4 (4.6%)	
		GCSE more than a C	6 (7.8%) 7 (9.1%)	20 (23%) 14 (16.1%)	
		A levels	10 (13%) 5 (6.5%)	11 (12.6%) 15 (17.2%)	
		HND	11 (14.3%) 11 (14.3%)	22 (25.3%) 16 (18.4%)	
		Degree	41 (53%) 42 (54.5%)	23 (26.5%) 21 (24.1%)	
5. *When your child is asleep how often do you nudge/touch them to make them breath again?*	Smokers in the house (0.03)	Yes	9 (6.7%)	10 (24.4%)	0.003
		No	125 (93.3%)	31 (75.6%)	
	Parental education	Unknown	5 (3.8%) 12 (9%)	0 (0%) 7 (17%)	0.001 0.001
		No examinations	5 (3.8%) 4 (3%)	3 (7.3%) 5 (12%)	
		GCSE less than a D	7 (5.3%) 8 (6%)	2 (4.9%) 2 (4.9%)	
		GCSE more than a C	12 (9%) 11 (8.3%)	14 (34.1%) 9 (22%)	
		A levels	15 (11.3%) 15 (11.3%)	8 (19.5%) 6 (14.6%)	
		HND	25 (18.8%) 20 (15%)	7 (17.1%) 8 (20%)	
		Degree	64 (48.%) 63 (47.4%)	7 (17.1%) 4 (9.5%)	
7. *How often does your child have restless sleep?*	Use of prophylactic asthma treatment	Yes	2 (4.%)	23 (17.7%)	0.018
		No	16 (32.7%)	51 (39%)	
		Not applicable	31 (63.3%)	56 (43.3%)	
	BMI category	Normal	0 (0%)	4 (4.1%)	0.022
		Underweight	13 (65%)	79 (81.4%)	
		Overweight	4 (20%)	2 (2.1%)	
		Obese	3 (15%)	12 (12.4%)	
8. *How often does your child sweat while asleep?*	Presence of smokers	Yes	5 (25%)	97 (62.6%)	0.002
		No	15 (75%)	58 (37.4%)	
	Presence of wheeze	Yes	43 (42.2%)	43 (58.9%)	0.025
		No	59 (57.8%)	29 (39.7%)	
		Not applicable	0 (0%)	1 (1.4%)	
	Parent 1 education level	Unknown	4 (3.9%)	1 (1.4%)	0.028
		No examinations	3 (2.9%)	6 (8.3%)	
		GCSE less than a D	3 (2.9%)	6 (8.3%)	
		GCSE more than a C	12 (11.8%)	15 (20.8%)	
		A levels	14 (13.7%)	8 (11.1%)	
		HND	16 (15.7%)	16 (22.2%)	
		Degree	50 (49.1%)	20 (27.9%)	
10. *How often does your child have difficulty waking up in the morning, even after getting plenty of sleep?*	Parent 2 education level	Unknown	11 (13.5%)	9 (30%)	0.003
		No examinations	6 (7.4%)	3 (10%)	
		GCSE less than a D	8 (9.9%)	2 (6.7%)	
		GCSE more than a C	18 (22.2%)	2 (6.7%)	
		A levels	16 (19.8%)	7 (23.3%)	
		HND	22 (27.2%)	7 (23.3%)	

Further clarification of educational level: 1. GCSE at level C: has passed examinations conducted at the age of 16 years, 2. A-levels: has obtained examination results at the age of 18 years, 3. HND: post-18 year higher education achievement taken as alternative to a degree for more vocational subjects, 4. Degree: successful completion of a university course

*For asthma treatment, this was only recorded for children who reported a history of wheeze

**For parental education the first parent is shown in black and second parent in gray.

### Psychometric Properties

A principle component analysis was conducted on 13 items with an orthogonal rotation (varimax). The Kaiser-Mayer-Olkin (KMO) measure verified the sampling adequacy for the analysis with a KMO =0.843, additionally all individual KMO values were above the acceptable limit.

Initial analysis extracted three factors with Eigen-values greater than 1. This explained 61.7% of the variance, however most of the variance was accounted for by the first factor (41.3%). The screen plot showed inflexion both at the second factor and the 4^th^, with the 3^rd^ and 4^th^ factor contributing a similar amount of the total variance. To interpret the factors further it was forced to produce either a two, three, or four factor solution. These were reviewed by the authors and it was felt that a four-factor solution was the most appropriate and meanings were assigned to each factor, generating subscales where factor 1 represented breathing and physical related symptoms; factor 2 represented night time behavior; factor 3 represented morning behavior; and factor 4 represented the impact of poor sleep on the next day’s behavior. Items were considered to load on to a factor if they had a value of greater than 0.3 and substantially load if they had value greater than 0.7. If items loaded onto multiple factors they were assigned to the factor in which they had the highest loading. If they had similar loading, as was the case with item 5 (when your child is asleep, how often do you touch/nudge your child to make them breathe again), they were assigned to the factor which clinically matched the best item. Loading of the factors determined the sub-scale structure of the questionnaire which is shown in **Table 4**.

**Table 4:** Structure of the questionaire.

**Breathing Subscale**	
	How often does your child snore when they do not have a cold?
	How often can you hear your child snoring from outside the bedroom?
	How often can you hear your child struggle to breathe while asleep?
	How often does your child’s breathing go quiet and then he, she gasp?
	How often does your child sweat while asleep?
**Night time behavior subscales**	
	How often does your child have restless sleep?
	How often does your child wake up during the night (compare to a child of a similar age)
	How often does your child sleep in unusual position?
**Morning behavior subscale**	
	How often does your child have difficulty waking up in the morning even after getting plenty of sleep?
	How often is your child grumpy first thing in the morning?
**Impact of poor sleep on next day behavior subscale**	
	How often is your child unusually sleepy during the day?
	How often does your child appear more hyperactive or fidgety than children of a similar age?

#### Reliability

A split half method was used to examine the internal consistency of the individual subscales. Spearman Brown coefficients were 0.8 for subscale 1, 0.79 for subscale 2, 0.75 for subscale 3, and 0.5 for subscale 4. An acceptable reliability was therefore achieved in subscales 1–3 but not subscale 4.

The reliability for the scale, as a whole, was assessed using Cronbach alpha which gave a value of 0.87

### Receiver Operator Characteristic Analysis

The mean and standard deviation for the total score and subscale scores are shown in **Table 5**.

**Table 5:** Questionaire total score and subscales scores.

	Mean score (SD)
Total score	33.3 (10)
Breathing subscale	14.1 (5.6)
Night-time behavior subscale	10.7 (3.8)
Morning behavior subscale	3.5 (1.7)
Impact of poor sleep on the next day behavior subscale	5.1 (2.02)

The AUC for the total questionnaire score was 0.497 (95% CI 0.352–0.642) for on OAHI > 5/h, and 0.569 (0.360–0.778) for an OAHI > 10/h. The breathing subscale gave an AUC of 0.542 (0.407–0.677) for an OAHI> 5/h, and 0.603 (0.409–0.796) for an OAHI> 10/h. AUC values for other subscales are shown in **Table 6**. ROC analysis was additionally performed for the other subscales and is shown in **Table 6**. Furthermore, the group was stratified by gender, age, and previous ear, nose, and throat (ENT) surgery for the total score and breathing subscale. No AUC greater than 0.7 was achieved.

**Table 6:** Area under the curve values for total score of the questionnaire and subscales for obstructive apnea hypopnea index (OAHI).

	OAHI≥5 (95% CI and standard error)	OAHI≥10 (95% CI and standard error)
Total questionnaire score	0.497 (0.352–0.642) SE: 0.074	0.569 (0.360–0.778) SE: 0.107
Breathing and physical related symptoms subscale	0.542 (0.407–0.677) SE: 0.069	0.603 (0.409–0.796) SE 0.099
Night time behavior subscale	0.307 (0.234–0.506) SE: 0.070	0.442 (0.233–0.651) SE 0.107
Morning behavior subscale	0.501 (0.354–0.647) SE: 0.075	0.596 (0.403–0.790) SE: 0.099
Impact of poor sleep on the next day behavior subscale	0.618 (0.48–0.755) SE: 0.070	0.663 (0.490–0.836) SE: 0.088

It was possible that some meaningful data were lost from over-stringent removal of questionnaires with unsure responses. Therefore, factor analysis was repeated including these questionnaires and replacing unsure responses with a mean imputation method. There was no change in the factors extracted. Next, including this complete data set, ROC curve analysis was repeated. The AUC for the total score to predict OAHI > 5/h was 0.515. Further analyses were not performed.

#### Predictive Value

**Figure 2:**
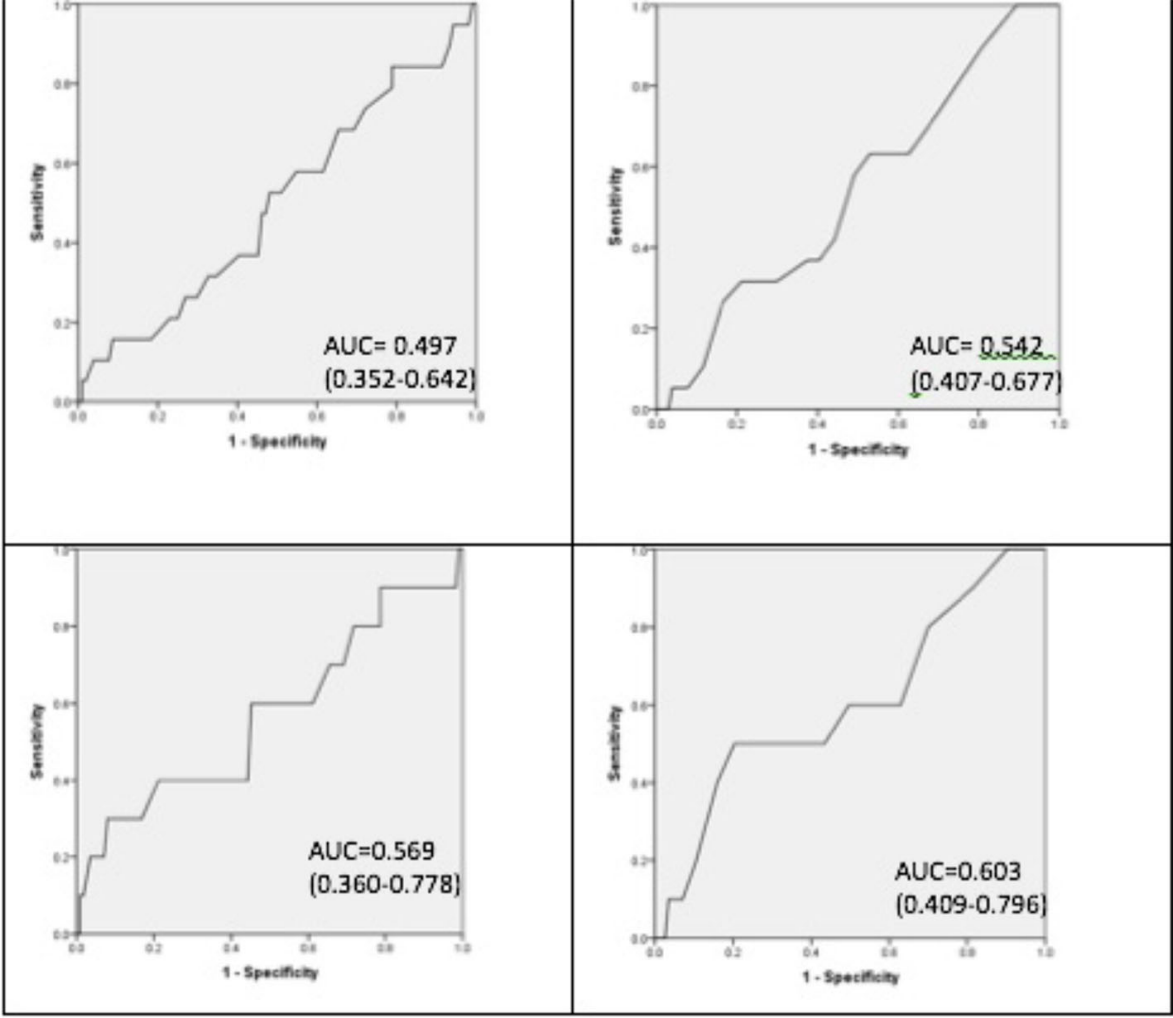
Receiver operating characteristic curves. From left to right. Top left obstructive apnea hypopnea index (OAHI)≥5/h and whole questionnaire score. Top right OAHI > 5/h and breathing subscale. Bottom left OAHI≥10/h and whole questionnaire score. Bottom right OAHI≥10/h and breathing subscale.

Based on ROC curve analysis to maximize sensitivity an optimal total questionnaire score cut off score of 19.5 (out of a total of 65) was generated. This identified 18/19 of the true positives (sensitivity of 94.7%) and 6/104 of true negatives (specificity of 1.9%). The positive predictive value was 0.14 and negative predictive value was 0.86. This is illustrated in **Figure 2**, which highlights the failure of the questionnaire to screen out children with OAHI ≥ 5/h. The predictive value of the questionnaire did not improve when the 22 children with a past history of upper airway surgery were removed from the sample. In practice, therefore, for every 100 children screened, 94 would screen positive and require confirmatory diagnostics.

## Discussion

We have demonstrated that the DS OSA questionnaire has poor positive predictive value for clinically relevant OSA in young children with DS, despite robust psychometric properties. This supports previous findings that parental report in children with DS is a poor predictor of OSA (14, 37–39).

Similarly, the literature indicates that health professionals struggle to diagnose OSA based on clinical findings in DS patients, even when supported by questionnaire items (13, 29, 40). Recent data from the UK support our findings by demonstrating that the PSQ questionnaire, which has established high sensitivity in TD children, performs very poorly in this group (31).

Other groups have combined simple objective measures, such as BMI, with questionnaire data and medical history to improve prediction of OSA. Skotko et al. developed a tool to identify OSA in the Down syndrome population using data from 130 patients aged 3–24 years (22). The model had 300 rules and 101 variables, including questions from the CSHQ and sleep-related breathing disorder subscale of the PSQ questionnaires, patients’ past medical history, physical examination, and BMI. This model had a negative predictive value of 90% and positive predictive value of 25% for an AHI ≥5/h. While this shows promise it has yet to be validated in another data set. Furthermore, given the large number of variables required for analysis, it may not be a simple tool to introduce into routine clinical practice unless technological aids are also established (22).

Development of the DS OSA questionnaire closely followed recommended methodology (33). The failure of this questionnaire to be a useful screen for OSA, despite a structured design process and good psychometric properties, reminds researchers of the importance of objective screening measures for OSA in clinical practice. It also serves to remind clinicians about the importance of only using questionnaire tools that have been robustly validated in the relevant population.

A key limitation of the questionnaire was the inclusion of the unsure response item. The aim was to prevent respondents giving false response to questions. It also allowed us to identify questions which could potentially lack clarity. This did, however, result in the exclusion of 33.9% of the sample. Given that there were no significant demographic or clinical differences between the final sample and the excluded group it is unlikely that this led to any systematic bias. Furthermore, using mean imputation methods to replace these questions did not change the factor structure of the questionnaire.

A further limitation of our data was the use of cardiorespiratory polygraphy rather than gold standard polysomnography to generate the OAHI. Cardiorespiratory studies tend to underestimate the OAHI as this technique cannot detect hypopneas associated with arousal. Use of cardiorespiratory studies in our study was a pragmatic choice reflecting typical UK practice. Furthermore, recent data in children indicate that this technology predicts OSA (defined by OAHI ≥ 5.6/h from polysomnography) with a sensitivity of 90.9% (95% CI, 79.6–100%) and a specificity of 94.1% (95% CI, 80–100%) (41). For this reason, we selected an OAHI of >5/h as a threshold to define OSA resulting in a prevalence rate of OSA in the sample of only 15%.

An additional limitation was that measurements for height and weight were only taken once by trained research nurses. A single measure may have led to inaccuracy.

Higher prevalence rates of OSA have been reported in large sample of individuals with DS. However, prevalence rates are influenced by age, sampling strategy, and the threshold used to define OSA. For example, Maris et al. reported OSA in 66.4% of 122 children with DS aged 0–18 years (based on a threshold of OAHI of >2/h) (42). However, 57% of these children were clinically referred with concerns about apnea. In contrast Skoto et al. reported lower rates of 44.4% in 56 children aged 3–5 years randomly selected from a DS follow-up program at Boston Children’s Hospital (based on a threshold of OAHI of >2/h) (22). Due to the lack of state funded healthcare in the USA it is possible that children with access to regular care were from wealthier families. In the same way, however, social class can influence clinical research participants. Indeed in our study 42% of children had a parent who was a graduate suggesting a similar class bias in both studies (43). Our study population had a narrow age range (0.5–6 years), were largely community recruited and we used a threshold OAHI of > 5/h to reflect the sensitivity of cardiorespiratory polygraphy for the present analysis. Using a threshold of OAHI >2/h in our sample prevalence rates of OSA are 46.3%, almost identical to the Skoto figures, although there are difference as noted above in the population recruited. Also of note 17.8% of the final sample of 123 children had previously had adenotonsillectomy, potentially reducing their OAHI.

**Figure 3:**
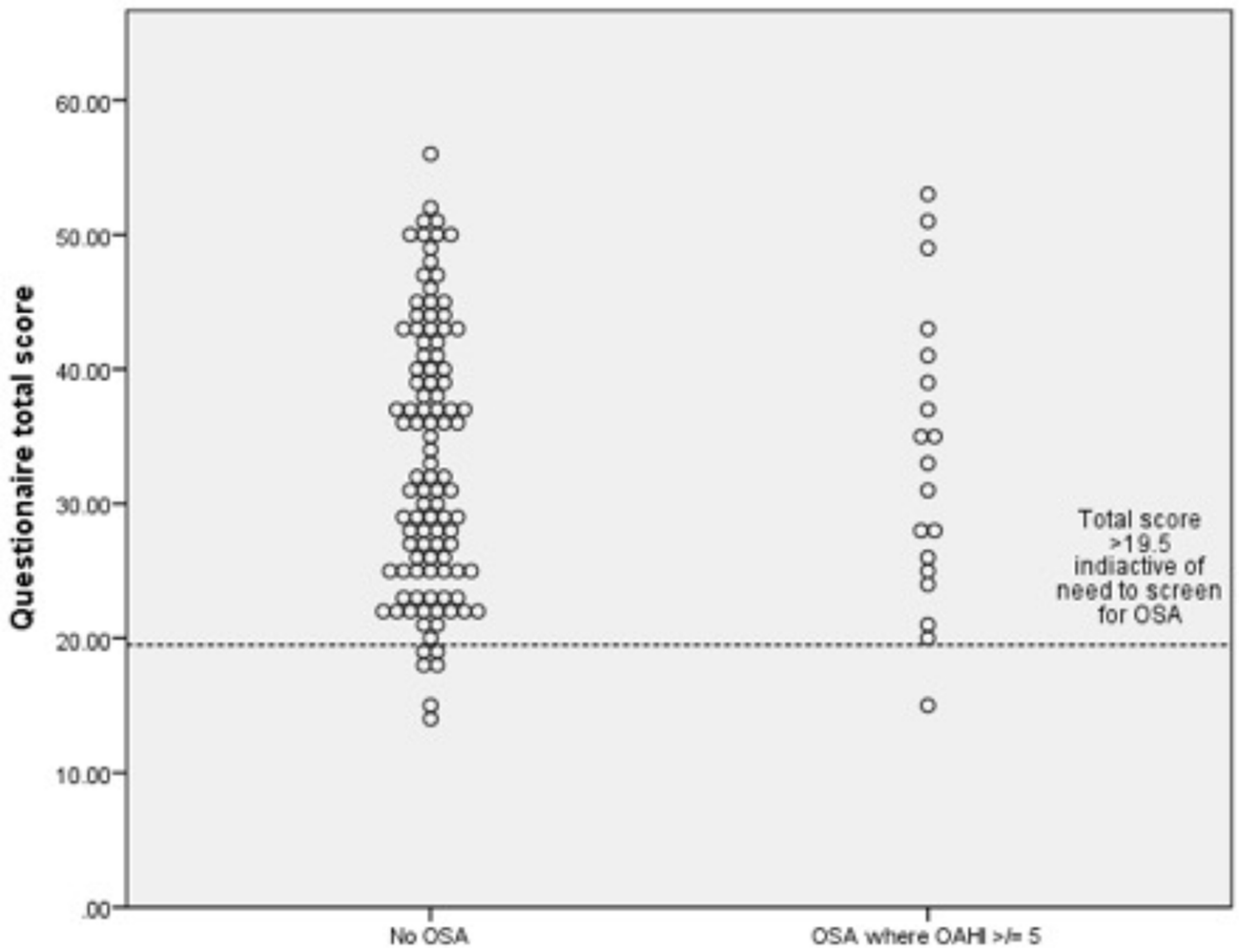
Dot plots for obstructive apnea hypopnea index (OAHI) for (n-123) for children with and without obstructive sleep apnea (OSA) with a questionnaire score cut off of 19.5.

While in principle the low numbers with OSA as defined in this study may have reduced our ability to explore the validity of the questionnaire, as illustrated by **Figure 3**, there were no differences in responses between those with and without OSA.

The research field attests to a motivation of clinicians and researchers to offer a simpler screening alternative to cardiorespiratory or polysomnographic evaluation of OSA for children with DS. This motivation is understandable as sleep studies are expensive and may be poorly tolerated by children with learning disabilities. Alternative screening methods have been researched to offer a non-invasive alternative to polysomnography. Esbensen et al. investigated the potential of actigraphy to identify OSA in 27 children aged 5–17 years with DS. Actigraphy correlated with PSG for the total sleep time, wake after sleep onset, and sleep efficiency but not a clinical diagnosis of OSA (44). Elsharkawi et al. reported that a combination of four urinary biomarkers had a positive predictive value of 90% and negative predictive value of 68% to predict OSA at an AHI≥1/h (21). These techniques are expensive, not widely available and the authors noted that further studies were required in larger populations before this approach could be recommended as a screening tool. Imaging techniques have been studied. Three-dimensional photogrammetric measurements have been compared in DS children with and without OSA and with no differences established (20). Similarly, cephalometry was not found to usefully contribute to prediction of OAHI in a study of 130 children and young adults with DS (22). UK Royal College of Paediatrics and Child Health currently recommends screening children with DS annually for OSA from infancy to 5 years with a minimum of pulse oximetry (19). There has previously been little evidence to support this technique in this population but we have recently demonstrated a high sensitivity (92%) and specificity (63%) of one night of domiciliary Masimo pulse oximetry to predict OSA diagnosed by cardiorespiratory polygraphy (18).

## Conclusion

A carefully constructed questionnaire with good content validity lacks criterion validity to make it a useful tool in clinical practice. This is in keeping with the literature that parental report and clinical evaluation in routine practice are poor predictors of OSA in DS. As such, objective screening methods should be adopted and our previous findings suggest that domiciliary pulse oximetry could offer an acceptable first-line screening approach, halving the number of children requiring more detailed sleep studies.

## Data Availability Statement

The datasets generated for this study are available on request to the corresponding authors.

## Ethics Statement

The studies involving human participants were reviewed and approved by UK National Research Ethics Committee (reference 13/SC/0106). Written informed consent to participate in this study was provided by the participants’ legal guardian/next of kin.

## Author Contributions

CH and HJE conceived the idea for the study. CH, HEE, CT, and ES designed the questionnaire. CH, HJE, HEE, RK, JM, JR, AJ, and PG were involved in the recruitment of the subjects, conducting the questionnaire and PSG. SG-H, CH, and KS were involved in data analysis, data interpretation, and authoring the manuscript.

## Funding

This study was supported by Action Medical Research and the Garfield Weston Foundation [grant reference 2040].

## Conflict of Interest

The authors declare that the research was conducted in the absence of any commercial or financial relationships that could be construed as a potential conflict of interest.
